# Marker-Assisted Selection for Pollen-Free Somatic Plants of Sugi (Japanese Cedar, *Cryptomeria japonica*): A Simple and Effective Methodology for Selecting Male-Sterile Mutants With *ms1-1* and *ms1-2*

**DOI:** 10.3389/fpls.2021.748110

**Published:** 2021-10-12

**Authors:** Momi Tsuruta, Tsuyoshi E. Maruyama, Saneyoshi Ueno, Yoichi Hasegawa, Yoshinari Moriguchi

**Affiliations:** ^1^Department of Forest Molecular Genetics and Biotechnology, Forestry and Forest Products Research Institute, Tsukuba, Japan; ^2^Graduate School of Science and Technology, Niigata University, Niigata, Japan

**Keywords:** clonal propagation, Cupressaceae, gibberellin-induced flowering, loop-mediated isothermal amplification diagnosis, male-sterile plant, *MS1* gene, PCR marker, somatic embryogenesis

## Abstract

Pollen allergy caused by sugi (Japanese cedar, *Cryptomeria japonica*) is a serious problem in Japan. One of the measures against pollinosis is the use of male-sterile plants (MSPs; pollen-free plants). In this context, the development of a novel technique for the efficient production of sugi MSPs, which combines marker-assisted selection (MAS) with somatic embryogenesis (SE), was recently reported by our research group. To improve the efficiency of MSP production, in this paper we report improved MAS for male-sterile individuals from embryogenic cells, cotyledonary embryos, and somatic plants of sugi using a newly developed marker in the form of the causative mutation of *MS1* itself, selecting individuals with *ms1-1* and *ms1-2* male-sterile mutations. We also describe simplified methods for extracting DNA from different plant materials and for MAS using LAMP diagnostics. Finally, we show that MAS can be efficiently performed using the one-step indel genotyping (ING) marker developed in this study and using InstaGene for DNA extraction. The combination of SE and 100% accurate marker selection during the embryogenic cell stage enables the mass production of *MS1* male-sterile sugi seedlings.

## Introduction

Tree pollen-induced allergy has been reported in many countries around the world, and currently almost 40% of people living in Japan suffer from pollinosis caused by sugi [Japanese cedar, *Cryptomeria japonica* (Thunb. ex L.f.) D.Don, Cupressaceae] pollen (Matsubara et al., 2020). As a countermeasure against this, there is an urgent need to spread sugi cultivars with low pollen production or male sterility. Sugi is the most commercially important forestry conifer in Japan, where it covers approximately 4.4 million ha (44% of the total artificial forest; Forestry Agency, 2020). Replacing these trees planted on a vast scale with low-pollen-producing or male-sterile plants (MSPs; pollen-free plants) would require many seedlings, but is also associated with other problems.

First, to date, 23 strains of male-sterile sugi have been found (Saito, 2010), but the materials that can be used for breeding are still limited. *MALE STERILITY* (*MS*)*1* to *MS4* are known as the causative genes of male sterility in sugi, named after the different abnormalities in pollen development with which they are associated (Taira et al., 1999; Yoshii and Taira, 2007; Miyajima et al., 2010; Saito, 2010). Of these, the major gene for male sterility is *MS1*, associated with a developmental abnormality in the pollen tetrad period, caused by one recessive allele (*ms1*; Taira et al., 1999; Miura et al., 2011). Recently, Hasegawa et al. (2021) identified the *MS1* gene itself, determining that the causative mutation was a 4- or 30-bp deletion in the coding region of the CJt020762 gene, designated as *ms1-1* and *ms1-2*, respectively. Using the mutations as genetic markers, it is now possible to accurately identify male-sterile (homozygous for *ms1*) and potentially sterile trees (heterozygous for *ms1*), as well as male-fertile trees (wild-type), making it easier to search for material that can be used for sugi breeding (Hasegawa et al., 2020, 2021; Moriguchi et al., 2020). Of these trees with *ms1* alleles, *ms1-1* (i.e., 4-bp deletion) is the major causative mutation, whereas only seven trees with an *ms1-2* mutation (30-bp deletion) allele were found in previous screenings (Moriguchi et al., 2020; Hasegawa et al., 2021).

Another major problem is the mass propagation of seedlings. Currently, male-sterile sugi seedlings are eventually obtained after the artificial crossing of a sterile tree (*ms1*/*ms1*) as a seed parent and a pollen donor with heterozygous sterile alleles (*Ms1*/*ms1*), growing seedlings from the seeds, inducing male flowering by the application of gibberellin (GA), and then selecting MSPs. Therefore, about half of the obtained plants are unusable fertile individuals and maintaining those strains until male-sterile and -fertile plants can be selected is extremely resource-intensive in terms of labor and space. Thus, our goal is to establish protocols for efficiently propagating male-sterile sugi seedlings by combining early selection of pollen-free lines using genetic markers (marker-assisted selection or marker aided selection, MAS) and a clonal mass propagation method. Among the available clonal propagation methods, somatic embryogenesis (SE) is the most attractive technique for the large-scale propagation and long-term conservation of different embryogenic cell lines (ECLs) without changing their initial characteristics (Park et al., 1998). However, for many coniferous species, *in vitro* clonal propagation is still difficult or inefficient (Bonga et al., 2010). Currently, the most well-established source of SE propagation in sugi is immature zygotic embryos, for which protocols have been established up to the stage of plant regeneration (Maruyama et al., 2020, 2021a, 2021b). Selecting a male-sterile strain as early as possible in this process will improve the efficiency of MSP production. In a study by Maruyama et al. (2020), a marker closely linked to *MS1* was used to identify male-sterile ECLs.

In the present study, we aimed to further improve the efficiency of MAS for a system for effectively propagating sugi by focusing on the following three points. (1) Examination of genetic markers to be used: The linked marker used in the previous study was not the *MS1* gene itself, but closely linked to *MS1* (0.58cM; Ueno et al., 2019). This genetic distance means that recombination can occur in 1 out of 200 individuals, and they can be misclassified (Ueno et al., 2019). Additionally, linked markers need to be carefully evaluated in terms of whether they can be adapted to different families from the one in which the marker was developed. Therefore, in this study, we used direct genetic markers that are markers of the causative mutation of the *MS1* gene itself for MAS. Allele-specific PCR (ASP) markers, which detect each wild-type (*Ms1*) or mutated allele (*ms1-1*), were developed on the causative CJt020762 gene (Hasegawa et al., 2020). We also developed a new genetic marker, named the single-tube indel genotyping (ING) marker, which can immediately determine the genotype of the causative gene, which can improve the accuracy of selection. The detection of male sterility by loop-mediated isothermal amplification (LAMP, Notomi et al., 2000), in which the reaction is completed faster than for PCR (typically within 1h) and there is no need for electrophoresis, is expected to lead to further time savings. LAMP applicability for MAS of MSP was also investigated.

(2) We also investigated DNA extraction methods to save labor. Currently, a variety of DNA preparation methods and commercial kits are available. Of these, freeze-grinding under liquid nitrogen (LN_2_) and extraction with hexadecyltrimethylammonium bromide (CTAB) buffer are commonly used for sugi, but they are time- and labor-intensive (e.g., Maruyama et al., 2020; Moriguchi et al., 2020; Hasegawa et al., 2021). Simple DNA extraction methods that do not require grinding with LN_2_ and toxic chemicals like phenol and chloroform, such as using Chelex resin and filter paper, have been reported in not only model plants and grasses but also some woody plant species (Berthold et al., 1993; Lange et al., 1998; Lin et al., 2000; HwangBo et al., 2010; Siegel et al., 2017). For MAS of a large number of samples, these simple DNA extraction methods will be effective (Mbogori et al., 2006). The applicability of these methods was verified at each stage of SE. Finally, (3) by combining these methods, we investigated which culture stages and methods were suitable for *MS1* diagnosis. From the above, we provide an effective MAS protocol on male-sterile sugi SE propagation that will be applicable to conifer SE breeding.

## Materials and Methods

### Plant Material and Culture Conditions for SE Initiation

Embryogenic cells (ECs), somatic embryos, and plants derived from five full-sib seed families of *C. japonica* carrying the male-sterile allele in *MS1*, namely, *ms1*, were used as the main experimental materials for this study (*N*=93, Table 1). Additionally, plant materials of four lines derived from seed families without any male-sterility alleles (T2-14-1, T2-14-149, T4-4-1, and T4-11-51) were used as an experimental control. For SE initiation, the entire megagametophyte (~3–4mm long) including zygotic embryo was used as the initial explant. The collected seeds were surface-sterilized with 1% (w/v) antiformin solution (Sodium Hypochlorite Solution; Wako Pure Chemical, Osaka, Japan) for 15min and then rinsed three times with sterile distilled water for 5min each, before isolation of megagametophyte explants. For the induction of ECs, explants were placed horizontally onto initiation medium contained in 90×15-mm quad-plates (3 explants per well, 12 per plate) sealed with Parafilm® and cultured in the dark at 25°C. Initiation medium containing basal salts reduced by half the concentration from the standard EM medium (Maruyama et al., 2000) was supplemented with 10gL^−1^ sucrose, 10μM 2,4-dichlorophenoxyacetic acid (2,4-D), 5μM 6-benzylaminopurine (BA), 0.5gL^−1^ casein acid hydrolysate, and 1gL^−1^ glutamine, and solidified with 3gL^−1^ gellan gum. The pH was adjusted to 5.8 prior to autoclaving the medium for 15min at 121°C.

**Table 1:** Sugi seed families carrying the male-sterility gene *MS1* used as main experimental plant material.

Seed family	Male sterility genotype	Collection year	Reference^1^	Abbreviation name	Sample number
(‘Toyama-funen 1’×‘Ohara 2’) ×‘Suzu 2’	*Ms1*/*ms1-1* × *Ms1*/*ms1-1*	2014	[1]	TOS	3
‘Shindai 3’×‘Suzu 2’	*ms1-1*/*ms1-1* × *Ms1*/*ms1-1*	2016 2017	[2],[3] [2],[3]	S3S2 SS	39 47
‘Fukushima-funen 1’×(‘Shindai 3’×‘Kamikiri 2’)	*ms1-1*/*ms1-1* × *Ms1*/*ms1-1*	2017	[2],[3]	FSKam	10
‘Fukushima-funen 1’×‘Ōi 7’	*ms1-1*/*ms1-1* × *Ms1*/*ms1-2*	2017	[2],[3]	FO7	8
‘Fukushima-funen 1’×(‘Shindai 3’×‘Kashiwazaki-shi 3’)	*ms1-1*/*ms1-1* × *Ms1*/*ms1-1*	2017	[2],[3]	FSKas	6

1 Reference; ^[1]^ Maruyama et al. (2014), ^[2]^ Maruyama et al. (2020), ^[3]^ Maruyama et al. (2021a).

Mature seeds were also collected from the ‘Shindai 3’×‘Suzu 2’ family and seedlings were cultivated (*N*=20).

### Maintenance and Proliferation of ECLs

Tissues of established ECLs were regularly subcultured every 2–3weeks on maintenance/proliferation medium containing basal salts reduced to half the concentration from the standard EM medium (Maruyama et al., 2000) supplemented with 3μM 2,4-D, 1μM BA, 30gL^−1^ sucrose, 1.5gL^−1^ glutamine, and 3gL^−1^ gellan gum. Clumps of ECs (12 per plate) on plates sealed with Parafilm® were cultured in the dark at 25°C.

### Maturation of Somatic Embryos

For the maturation of somatic embryos, 2-week-old proliferated ECLs were cultured in clumps (5 masses per 90×20-mm plate, 100mg each) on maturation medium for 8weeks. Maturation medium containing the basal salt concentration of the standard EM medium (Maruyama et al., 2000) was supplemented with 30gL^−1^ maltose, 100μM abscisic acid, 2gL^−1^ activated charcoal, amino acids (in gL^−1^: glutamine 2, asparagine 1, arginine 0.5, citrulline 0.079, ornithine 0.076, lysine 0.055, alanine 0.04, and proline 0.035), 175gL^−1^ polyethylene glycol (Av. Mol. Wt.: 7,300–9,300; Wako Pure Chemical), and 3.3gL^−1^ gellan gum. The plates were sealed with Parafilm® and kept in the dark at 25°C.

### Germination, Plantlet Conversion, and Acclimation of Somatic Plants

Cotyledonary embryos picked up from maturation medium were laid horizontally onto germination medium (maintenance/proliferation medium containing 20gL^−1^ sucrose, 2gL^−1^ activated charcoal, and 10gL^−1^ agar, but without plant growth regulators) and cultured at 25°C under a photon flux density of 45–65μmolm^−2^ s^−1^ for 16h per day. The emergence of roots as “germination” and the emergence of both roots and epicotyl as “plant conversion” were recorded after 8weeks of culture. To promote the growth of converted plantlets, they were transferred to culture flasks containing growth medium (germination medium supplemented with 30gL^−1^ sucrose and 5gL^−1^ activated charcoal) and cultured under the same conditions as described above for about 10–12weeks before *ex vitro* acclimatization. Developed somatic plants removed from the culture flasks were gently washed with tap water to remove traces of agar from their roots and then transplanted into plant containers filled with Spagmoss (*Sphagnum subnitens*; Besgrow, Christchurch, New Zealand), and kept inside plastic boxes with transparent covers (Assist No. 2; Shinki Gosei Co., Ltd., Tokyo, Japan). Plant containers were irrigated with tap water as needed during the first 2weeks. After this initial 2-week period, the covers were gradually opened and the plant containers were fertilized with Nagao’s nutrient solution (Nagao, 1983). The covers were completely removed about 4weeks after transplanting. Subsequently, acclimated somatic plants were transplanted into individual Wagner’s pots (ICW-2; ICM Co., Ltd., Tsukuba, Japan) containing vermiculite and grown in a greenhouse for about 3months until an approximate height of 30cm was attained, after which they were treated with GA to induce male flowering.

### Induction of Male Flowering by GA

To induce male flowering in regenerated somatic plants of sugi, two spraying applications of GA were carried out during July and August. The first application was performed in late July (when the rainy season ends) and the second in early August (about 10days later). The treatment solution containing 100mgL^−1^ GA_3_ (Gibberellin A3; Wako Pure Chemical) was sprayed onto the somatic plants until dripping of the solution from the branches was evident. Treated somatic plants were fertilized twice a week with Nagao’s nutrient solution. To determine the presence or absence of normal pollen inside the flowers induced after treatment with GA_3_, the respective observations were performed on samples collected at the end of January. Randomly collected male flowers (about three to five per tested plant) were dissected longitudinally with a razor blade, after which they were observed under a stereomicroscope. The experiment was repeated in two consecutive years using at least three plants per tested line.

### DNA Extraction From ECLs, Cotyledonary Embryos, and Somatic Plants

**Figure 1:**
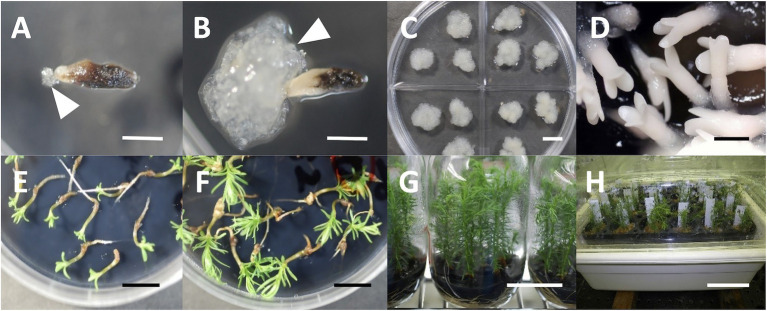
SE and plant regeneration in sugi (Japanese cedar, *Cryptomeria japonica*), **(A)** extrusion of ECs (arrow) from megagametophyte explant, **(B)** established ECL (arrow represent ECs), **(C)** maintenance/proliferation of ECs, **(D)** cotyledonary embryo formation from ECs, **(E)** embryo germination, **(F)** plantlet conversion, **(G)** *in vitro* growth of plantlets, **(H)** acclimation of somatic plants. *Bars*: 5mm **(A,B,D)**, 1cm **(C,E,F)**, 5cm **(G)**, and 10cm **(H)**.

From the ECLs (Figure 1C), cotyledonary embryos (Figure 1D), and shoots of developed plants (Figures 1G,H) and seedlings of 117 somatic plant lines consisting of five crossing families carrying the *ms1* allele (Table 1) and 4 non-*ms1* individuals (control), DNA was extracted using the simplified CTAB method and/or non-LN_2_ grinded extraction method described below. These samples had previously been genotyped for *MS1* using the *MS1*-linked marker (Maruyama et al., 2020).

From the shoot tips of *ex vitro*- or *in vitro*-cultured plants, crude genomic DNA was extracted using the simplified CTAB method. The pre-chilled sample with LN_2_ was powdered using TissueLyser II (Qiagen, Hilden, Germany), suspended in CTAB buffer (2% CTAB, 0.1M Tris–HCl, 20mM EDTA, 2M NaCl, 0.4% 2-mercaptoethanol, and 0.5% RNase A), and incubated at 65°C for 10min. An equal volume of chloroform/isoamyl alcohol (CIA) was added and mixed gently, and the supernatant was collected by centrifugation (11,000rpm, room temperature, 10min). This CIA isolation procedure was repeated twice. After adding a 3/4 volume of isopropanol and mixing gently, the nuclear precipitate was collected by centrifugation (15,000rpm, 4°C, 15min). After rinsing with 70% ethanol and drying, the DNA was dissolved in 200μl of nuclease-free water.

Thirty-one ECLs, 29 cotyledonary embryos, and 28 developed plant samples were used to investigate the applicability of the simple extraction method using InstaGene matrix (Bio-Rad, Hercules, CA, USA). The extraction procedure of InstaGene followed the method described by HwangBo et al. (2010). Briefly, 5mg of ECs, cotyledonary embryos, or a shoot tip of the developed plants was added to 200μl of InstaGene matrix without homogenization and vortexed for 10s. Following 8min of incubation at 100°C and vortexing for 10s, centrifugation was performed at 13,000rpm for 1min. The obtained supernatant was used as a DNA template. Some of these samples were also used for DNA extraction with a Whatman FTA MicroCard (GE Healthcare, Chicago, IL, USA). EC and cotyledonary embryo samples were applied to FTA Card by pressing the cells with a spatula. The shoot tip was applied by spotting 20μl of the homogenate ground in a mortar with 50μl of TE (10mM Tris-HCl, 0.1mM EDTA, pH 8.0). Then, each FTA Card was dried for at least 1–2h at room temperature. Subsequent DNA preparation, including disk sampling, washing with FTA Purification Reagent and TE buffer, and drying were performed in accordance with the enclosed manual.

### Development of One-Step Indel Genotyping Marker and 30-bp Deletion Diagnostic LAMP

Based on the strategy described by Lin et al. (2020), we developed a new *MS1* genotyping marker detecting the 4-bp deletion of *MS1* by single-tube PCR and agarose gel electrophoresis. Using the CJt020762 gene sequence of male-sterile and -fertile trees, ‘Fukushima-funen 1’ (*ms1-1*/*ms1-1*, GenBank ID: LC536580), ‘Ajigasawa 20’ (*Ms1*/*Ms1*, LC538204), and ‘Ōi 7’ (*Ms1*/*ms1-2*, LC538205), primer sequences were designed by Primer3 (Rozen and Skaletsky, 2000) and were selected to minimize the formation of self- and cross-dimers based on PrimerPooler (Brown et al., 2017). ‘Ōi 7’ was previously written as “Ooi-7,” but has been changed to follow the International Code of Nomenclature of Cultivated Plants (Brickell et al. 2016). The marker genotyping was verified using the male-fertile trees with different *MS1* genotypes, ‘Ōi 7’ (*Ms1/ms1-2*), ‘Suzu 2’ (*Ms1/ms1-1*), ‘Shimowada 29’ (*Ms1/Ms1*), and Ishinomaki 7 (*Ms1/ms1-2*), all of which had already been genotyped at *MS1* (Hasegawa et al., 2021). Ten microliters of PCR reaction mixture contained 1μl of DNA extract, 3μl of 2× QIAGEN Multiplex PCR Master Mix (Qiagen), and 0.2μM each primer. PCR cycling comprised denaturation at 94°C for 15min, followed by 35cycles of 94°C for 15s, 60°C for 30s, and 72°C for 30s, with final extension for 5min at 72°C. PCR products were then separated by 1–1.5% agarose gel (Agarose L03; Takara, Kusatsu, Japan) electrophoresis, stained with Midori Green Xtra (Nippon Genetics, Tokyo, Japan), and genotyped.

We also developed a rapid diagnostic primer set for the LAMP reaction to detect the 30-bp deletion of the *ms1-2* allele. Based on the allele sequences of *MS1* (‘Fukushima-funen 1’) and *ms1-2* (‘Ōi 7’), six primers (FIP, BIP, F3, B3, LF, and LB) were designed manually with reference to PrimerExplorer V5 software (Eiken Chemical, https://primerexplorer.jp/index.html). In some LAMP reactions, clamping primer of peptide nucleic acid (PNA) was used to suppress non-specific alternative allele amplification (e.g., Minnucci et al. 2012; Itonaga et al. 2016). We designed two PNA primers for the wild-type sequences corresponding to both ends of the 30-bp deletion. LAMP diagnosis was verified using ‘Ōi 7’ and ‘Suzu 2’ DNA. A total of 12.5μl of LAMP reaction mixture consisting of 1μl of template DNA, 6.25μl of WarmStart Colorimetric LAMP 2× Master Mix (New England Biolabs, Ipswich, MA, USA), 1.6μM each FIP and BIP primer, 0.2μMF3 and B3, 0.4μM LF and LB, and 1.6μM each PNA primer was incubated at 65°C for 90min. The color of the reaction solution was observed every 10min to determine the appropriate reaction time for the diagnosis of *ms1-2*.

### MAS for Male-Sterility Lines by PCR and LAMP Diagnostics

DNA extracted from each culture stage was used as a template to genotype *MS1* with genetic markers. The ASP marker detection for the presence of either wild-type or mutant allele *ms1-1* followed the procedure described by Hasegawa et al. (2020). For the ING marker, the reaction conditions were as described above, except that for the extracts of cotyledonary embryo and developed plant shoot by InstaGene and FTA Card, the reaction conditions were changed to 5μl of Multiplex PCR Master Mix and extension at 72°C for 45s. Hasegawa et al. (2021) showed that the male-fertile tree ‘Ōi 7,’ a heterozygous for *MS1*, had the *ms1-2* allele. In the offspring that had ‘Ōi 7’ as a pollen parent (FO7), we added amplified length polymorphism (ALP) marker genotyping (Hasegawa et al., 2020) to determine the presence of the *ms1-2* allele and the accurate *MS1* genotype. In addition, these FO7 samples and control individuals without *ms1-2* alleles were used to test the applicability of LAMP. The LAMP reaction was performed as described above, and the presence of an *ms1-2* allele was determined by the change in color (red to yellow) of the solution. In the case of FTA Card samples, the disk rinsed with nuclease-free water following TE washing steps was used as a template.

Finally, to choose the optimal genotyping method, the *MS1* phenotype, namely, male-sterile or -fertile, was compared among the previous *MS1*-linked marker results (Maruyama et al., 2020), observations of male flower dissections, and the genetic marker diagnoses of the present study. The genotyping results of ING marker and LAMP reaction were also compared between the templates of different cultured stages extracted by InstaGene and FTA Card.

## Results

### SE and Plant Regeneration

SE and plant regeneration of *C. japonica* were described in our previous reports (Maruyama et al., 2020, 2021a, 2021b). The extrusion of ECs from the explants could begin about 2weeks after the start of culture (Figure 1A) and the establishment of stable lines with evident EC proliferation was most frequently observed 4weeks after it (Figure 1B). Established ECLs can be maintained and proliferated by continuous routine subculture at 2–3-week intervals on maintenance/proliferation medium (Figure 1C). Embryo maturation was induced by culturing ECs on maturation medium (Figure 1D). Subsequently, germination (Figure 1E) and plantlet conversion (Figure 1F) from cotyledonary embryos were achieved about 1–2weeks and 3–6weeks after culture on plant growth regulator-free medium, respectively. Developed plantlets (Figure 1G) were successfully acclimated in plant containers (Figure 1H).

### Induction of Male Flowering

**Figure 2:**
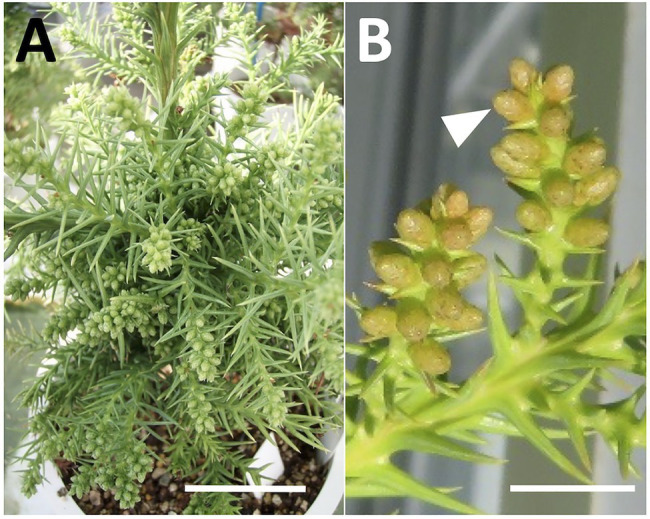
Induction of male flowering in sugi (Japanese cedar, *Cryptomeria japonica*) by GA application, **(A)** male flowering induction 2months after treatment, **(B)** male flower development 3months after treatment (arrow). *Bars*: 5cm **(A)** and 1cm **(B)**.

Male flowers were successfully induced after the application of GA_3_. The beginning of the formation of male flowers in the branches of the treated plants was observed approximately 1month after the second application of GA_3_ solution, showing evident development 2months after the treatment (Figure 2A). Subsequently, the flowers continued their development, reaching a size close to the maturity stage 3months after the GA_3_ treatment (Figure 2B). Then, when the flowers had fully matured (around late January), they were collected and dissected to confirm the presence or absence of pollen inside.

### Male Flower Collection and Dissection

**Figure 3:**
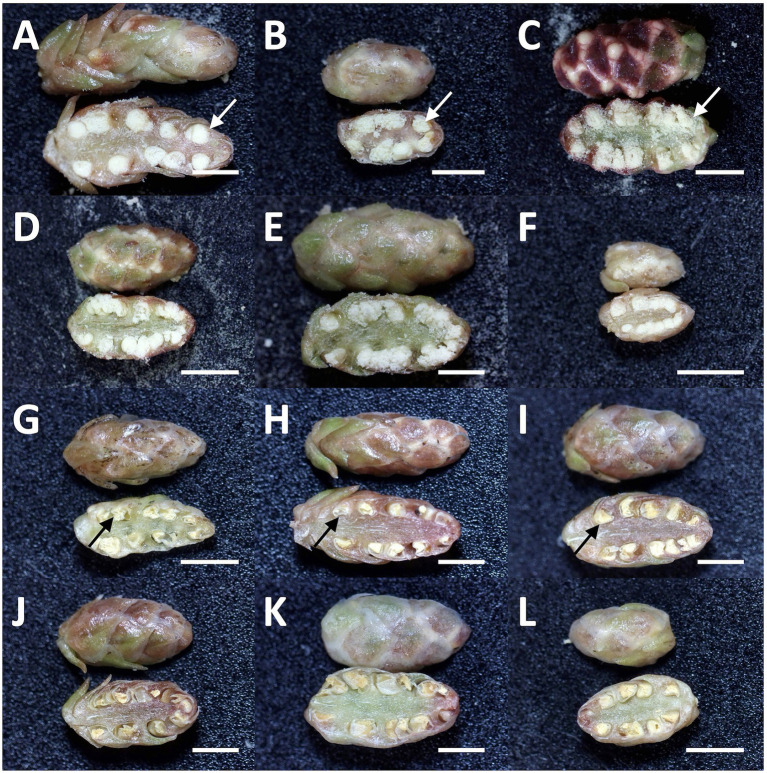
Representative types of dissected male flowers in sugi (Japanese cedar, *Cryptomeria japonica*) about 6months after GA application, **(A–F)** male flowers of male-fertile lines with normal pollen grains in the anthers (white arrow), **(G–L)** male flowers of male-sterile (pollen-free) lines without pollen grain (black arrow). *Bars*: 2mm.

Mutations in the *MS1* gene lead to the collapse of microspores after the separation of pollen tetrads (Saito et al. 1998). Therefore, we can observe full of pollen grains in anther locules in male-fertile trees, whereas yellow mass without pollen grains in male-sterile trees (Igarashi et al. 2006; Futamura et al. 2019). From the observation of dissected male flowers, we determined the male-fertile line or male-sterile line. The results from two consecutive years of observation are presented in Supplementary Table S1. Some representative types of male flowers in male-fertile and -sterile (pollen-free) lines are shown in Figure 3.

### Development of a Simple and Robust Indel Genotyping Marker

**Figure 4:**
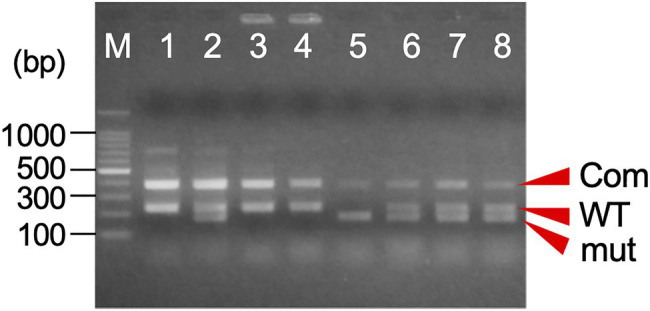
An example of the agarose gel electrophoresis of one-step indel genotyping marker for *MS1* diagnosis. The amplified PCR product consists of a common band (Com: 385bp+381bp) and the allele-specific band for wild-type (WT: 233bp) and/or mutant allele (mut: 183bp). M: 100bp ladder marker, 1: ‘O++i 7’ (*Ms1*/*ms1-2*), 2: ‘Suzu 2’ (*Ms1*/*ms1-1*), 3: ‘Shimowada 29’ (*Ms1*/*Ms1*), 4: Ishinomaki 7 (*Ms1*/*ms1-2*), and 5–8: somatic embryogenic lines.

Here, we have developed a simple genetic marker for determining the *MS1* genotype based on one of the causative mutations of male sterility, a 4-bp deletion in the CJt020762 gene (ING marker). The marker consists of two primer pairs specific for wild-type and mutant alleles (Table 2; Supplementary Figure S1). Two or three bands, namely, a common band (385bp and/or 381bp) and wild-type (233bp) and/or mutant-specific bands (183bp), were amplified by one-step PCR, and the genotype could be easily identified by agarose gel electrophoresis (Table 2; Figure 4). The genotype detection results corresponded with those of the previous *MS1* genotyping (Hasegawa et al., 2021).

**Table 2:** Primer sequences and products of one-step indel genotyping marker.

	Primer name	Sequence (5′ to 3′)	Amplified products	
A	Mt+rightPrimer_2_F	CTCACTGGCCACAGTCACAC	A+B: mutant allele specific band (183bp)	
B	Mt+rightPrimer_2_R	TGCAGGCAACTTATAATTAAGCAC		B+C: common band (385bp+381bp)
C	WT+leftPrimer_2_F	GACGTCTTCTGCAACAACAATGG	C+D: wild-type specific band (233bp)	
D	WT+leftPrimer_2_R	ACCCTGCGTGGGTGTTGATG		

### Identification of Male-Sterile Lines Using Genetic Markers

Using the ASP and new ING markers, we determined the *MS1* genotype in the 117 somatic plant lines, including 31 ECLs, 24 cotyledonary embryos, and 108 converted seedling samples (91 for ASP marker and 80 for ING marker, Supplementary Table S1). Of these, 45 lines were male-fertile and 72 were male-sterile. The detection results based on the ASP marker, ING marker, and male flower dissection for the 117 samples completely matched (Supplementary Table S1). The *MS1* genotyping results obtained in this study were almost entirely consistent with the previous results. Only the S-55 sample had different diagnostic results (Supplementary Table S1), which may have been due to an error in judgment resulting from recombination of the linked marker and the *MS1* locus. The *MS1* genotyping also required attention regarding the offspring of ‘Fukushima-funen 1’ and ‘Ōi 7’ (FO7). Here, by adding the genotyping of *ms1-2* using the ALP marker, the genotype of *MS1* could be accurately determined and corresponded with the male flower dissection results (Supplementary Table S1; Supplementary Figure S2A).

### PCR and LAMP Reaction With DNA Template From Different Extraction Methods

**Figure 5:**
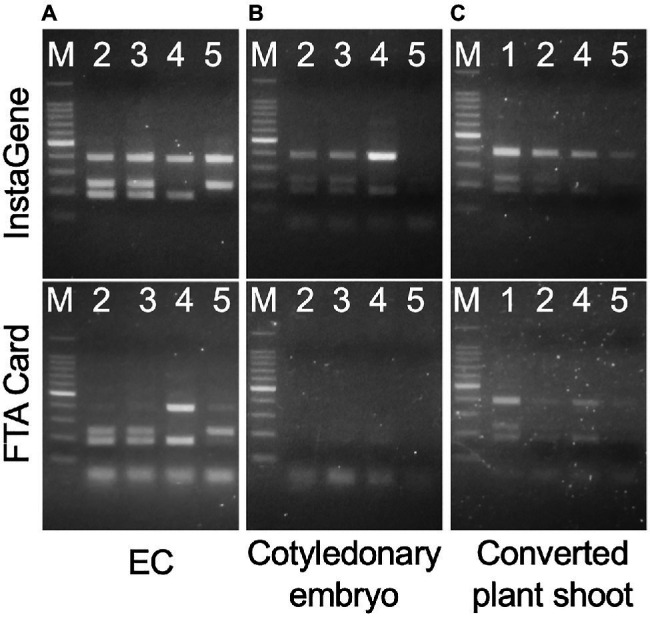
Amplification of one-step indel genotyping (ING) marker from EC **(A)**, cotyledonary embryo **(B)**, and developed plant shoot **(C)** extracted by InstaGene (top) and FTA Card (bottom). M: 100bp ladder marker, 1: FO7-97, 2: FO7-141, 3: FO7-144, 4: SSD-18, 5: T4-11-51.

Both the InstaGene and FTA Card extraction templates from the ECs enabled PCR amplification with the ING marker (Figure 5A), and the genotyping of *MS1* was the same as the results using DNA extracted from the seedlings as a template. In cotyledonary embryos, PCR amplification was also possible from InstaGene extracts. However, PCR amplification failed in some samples, and was never observed from FTA Card templates (Figure 5B; Supplementary Table S1). The extracted template of InstaGene and FTA Card from the shoots of developed plantlets could be applied to the MAS using the ING marker (Figure 5C). However, the amplified bands of the seedling samples tended to be somewhat weak in intensity.

A LAMP reaction system was constructed to detect alleles of *ms1-2* with the 30-bp deletion, including six primers and PNAs (Table 3; Supplementary Figure S1). After 30–40min of isothermal incubation at 65°C, the *ms1-2* allele was detected with a change in the color of the reaction solution (Supplementary Figure S2B). After 90min of incubation, the color change was also observed in control samples. Therefore, we chose the color change at 30–60min as an indicator of the presence of *ms1-2*.

**Table 3:** LAMP primers and PNA sequences for detecting alleles of *ms1-2* with the 30-bp deletion.

Primer name	Sequence (5′ to 3′)
FIP	ATGCAGTGATCCCCAAAATAGCCG-CGGATGATTCGCCCTTTCC
BIP	ACTGCATTGAAGTTTGATACACCG-TTGAACTCTGTTTCCATGGCA
F3	ATGGAGAGCGTTCCCGATC
B3	CGGAATACAGACAACAGCAAT
LF	GGCTCCAGCAATGCTAAC
LB	GAGGTTTAAAACTTTAACATATTTCTTC
PNA_WT1	GATCACTGCTAACCG
PNA_WT2	TTCAATGCAAATAAC

**Figure 6:**
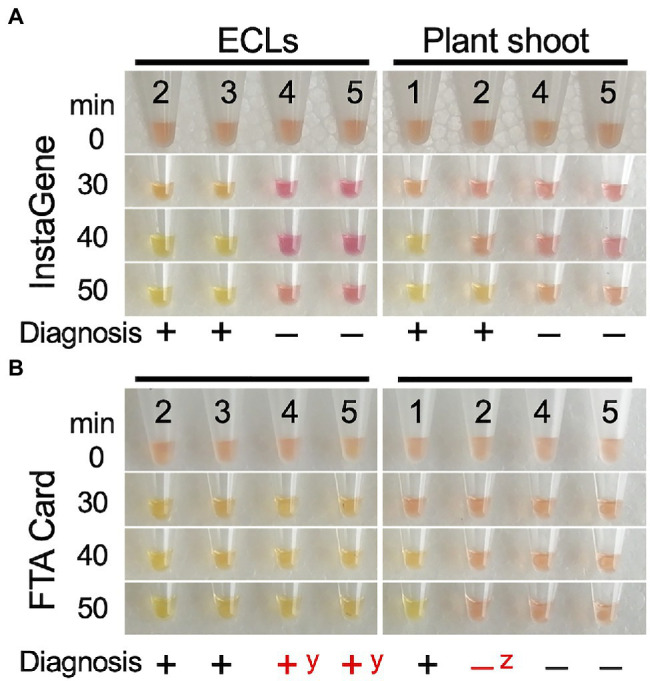
LAMP diagnosis of the presence of mutant allele *ms1-2* on the ECs and shoot tip of the developed plants using DNA template extracted by InstaGene **(A)** and FTA Card **(B)**. 1–3: *ms1-2*-positive samples (1: FO7-97, 2: FO7-141, 3: FO7-144), 4, 5: negative (without *ms1-2*) samples (4, SSD-18, 5: T4-11-51). ^y^false positive, ^z^false negative.

To test the adaptability of the LAMP reaction, DNA templates extracted by CTAB, InstaGene, and FTA Card in FO7 offspring were further used. For InstaGene extracts, the lines carrying the mutant *ms1-2* allele showed positive results after about 30min of isothermal reaction, while lines without *ms1-2* did not show any color change even after 60min of reaction (Figure 6A). LAMP diagnoses on both ECLs and developed plants were consistent with the *ms1-2* genotyping results of the ALP marker. However, shoot samples were often observed with a delayed reaction (taking 50min for color change, as shown in Figure 6A). FTA Card extracts of plant shoots also showed delayed responses and often false negatives (Figure 6B). Such false negatives were sometimes observed in the diagnosis with crude DNA of shoots extracted by the simplified CTAB method (Supplementary Figure S2B). In contrast, some FTA Card-extracted samples of plantlet shoots showed false positives (Figure 6B).

## Discussion

### Discrimination of Male-Fertile and -Sterile Lines Using Pollen Production After GA Treatment

The observations of male flowers for the discrimination of male-fertile and -sterile lines based on pollen production after GA treatment matched perfectly with our results of MAS using the mutation itself as a direct marker (Supplementary Table S1). This suggests that the discrimination of male-fertile and -sterile individuals based on the mutation itself as a direct marker is practically reliable and applicable for the early selection at the undifferentiated cell stage (EC) to produce MSPs at a rate of 100%. This implies that the GA-induced judgment method, which requires a lot of space and is labor- and time-intensive, can be replaced by the MAS methodology described in this paper. This would directly increase the efficiency of production of pollen-free plants and result in a low cost of seedlings (Table 4).

**Table 4:** MSP diagnosis methods used in this study and applicable SE materials.

Diagnostic methods	Detectable *MS1* allele (genotype)	Duration or reaction required for diagnosis	DNA extraction methods	Applicable SE materials^1^			References
				EC	Cotyledonary embryo	Developed plant	
Gibberellin-induced flowering+microscope	Fertile or male-sterile	3–4years	--	--	--	○	This study
*MS1* linked marker	*Ms1* and *ms1-1*	2 PCRs+electrophoresis	CTAB	○	n.d.	○	Ueno et al. 2019; Maruyama et al. 2020
ASP (allele-specific PCR) marker	*Ms1* and *ms1-1*	2 PCRs+electrophoresis	CTAB	n.d.	n.d.	○	Hasegawa et al. 2020; This study
ALP (amplified length polymorphism) marker	*Ms1* and *ms1-2*	One PCR+electrophoresis	CTAB	n.d.	n.d.	○	Hasegawa et al. 2020; This study
ING (one-step indel genotyping) marker	*Ms1* and *ms1-1*	One PCR+electrophoresis	CTAB InstaGene FTA Card	n.d. ○ ○	n.d. ▲ ×	○ ○ ○	This study
LAMP (loop-mediated isothermal amplification)	*ms1-2*	An isothermal reaction (less than 1h)	CTAB InstaGene FTA Card	n.d. ○ ×	n.d. n.d. n.d.	× ▲ ×	This study

1 ○: applicable, ▲: conditionally applicable, ×: unsuitable, n.d.: not done.

### MAS for Male-Sterile Lines Using Genetic Markers

Using the previously developed ASP marker and the newly developed ING marker, we were able to determine the genotype of *MS1* at three culture stages: ECs, cotyledonary embryos, and developed plants. In the allele-specific PCR including ASP (Hasegawa et al., 2020) and *MS1*-linked marker (Ueno et al., 2019), PCR amplifications were performed in separate tubes using each primer pair specific for the wild-type allele and the *ms1-1* mutant allele, and the genotypes were determined by the presence or absence of the specific band. If we are only interested in the presence or absence of one of the alleles, one reaction is sufficient, but to determine the genotype, it takes double the effort (Table 4). With the newly developed ING marker, the genotype of *MS1-1* could be easily determined with a single PCR and electrophoresis. In addition, the previous marker diagnosis was accompanied by difficulties in judgment with unclear PCR products (denoted as “doubted lines” by Maruyama et al., 2020), probably due to the instability of the band during PCR amplification. During our ASP marker detection, doubted non-specific amplification was also observed (data not shown). In addition, the previous decision was an erroneous judgment in one sample (S-55). This may have been due to so-called linked markers. Our newly developed ING marker was able to stably determine the *MS1-1* genotype and solved this problem. From the diagnosis using the mutation itself as a direct marker showing perfect agreement with the results of the male flower dissection, we would like to emphasize the importance of identifying the mutant gene itself for MAS in the breeding of *C. japonica*. This may now be referred to as “gene-assisted selection” or “molecular diagnosis.”

We also showed that determination of the *MS1* genotype requires attention on the family having different causative mutation sites on *MS1*. ‘Fukushima-funen 1’ is homozygous for the *ms1* allele caused by the *ms1-1* mutant allele (*ms1-1*/*ms1-1*), while ‘Ōi 7’ is heterozygous for the *ms1* allele derived from the *ms1-2* mutation (*Ms1*/*ms1-2*; Hasegawa et al., 2021). Therefore, the genotype of the offspring of FO7 would be *MS1*/*ms1-1* or *ms1-1*/*ms1-2*. Here, if only the genetic marker for the *ms1-1* mutation is used for discrimination, the genotype that is actually *ms1-1*/*ms1-2* would be mistyped as *Ms1*/*ms1-1*. The additional *ms1-2* determination would finally achieve a diagnosis that is consistent with the results of male flower dissection (Supplementary Table S1). Because heterozygous offspring of the two mutations in the coding region (*ms1-1*/*ms1-2*) became male-sterile, there is no doubt that the CJt020762 gene is *MS1* itself.

### Applicability of LAMP Reaction for Diagnosing *ms1-2*

We examined the applicability of the LAMP reaction for the diagnosis of *ms1-2* in the FO7 family. The lines carrying the mutant *ms1-2* allele showed positive results after about 30–40min of isothermal reaction. This is a significant time saving compared with PCR. Although the diagnosis results using the DNA template extracted from ECs by InstaGene were in complete agreement with those of the ALP marker, the reaction was delayed in some samples, potentially resulting in false-negative results, when shoot tips of the developed plants were used as the template. The same misjudgment was also observed when using crude DNA extracted from the seedlings, suggesting that the reason for this is not the yield of DNA, but rather the contaminants of secondary metabolites in the adult leaves as inhibitors of the enzymatic reaction. LAMP should be applicable to ECLs, which may contain fewer of these inhibitors, and FTA Card extraction could not be applied to the LAMP reaction (Table 4). Another limitation of LAMP is that it is necessary to add a wild-type diagnostic reaction in order to determine the genotype because LAMP is a dominant marker.

### Simple and Effective Combination of DNA Extraction and Marker Analysis for Achieving MAS of *MS1* in Somatic Plants

As recently reported, two mutations in the same *MS1* gene have been found to cause male sterility (Hasegawa et al., 2021). Therefore, in some crossing families, for example, FO7, it is necessary to determine both *ms1-1* and *ms1-2* mutation alleles in order to detect male sterility. To identify both *ms1-1* and *ms1-2* genotypes simultaneously, the method of ALP_ms1 reported by Hasegawa et al. (2020) can be used. This method is accurate and cost-effective (Moriguchi et al., 2020), but requires expensive laboratory equipment such as capillary sequencers. PCR and electrophoresis-based methods, such as ASP and our ING markers, do not require capillary sequencers and can be used for rapid determination. If in a less well-equipped laboratory, LAMP can also be used for *MS1* MAS, but it is limited for ECLs with the CTAB or InstaGene extraction methods (Table 4).

To increase the effectiveness of MAS, it is best to perform the diagnosis at an early stage of culture. In other words, it is expected to be applied to the initial ECLs. In this case, DNA extraction methods of both InstaGene and FTA Card were applicable for PCR markers. These methods yielded a DNA template with simple laboratory equipment from ECLs. Of these, FTA Card takes about half a day, including a few hours of drying, while InstaGene takes only 15min per sample. On the other hand, in later SE samples, cotyledonary embryos and shoot tips of developed plantlets, PCR amplification, and LAMP reactions become increasingly difficult. Sugi leaves contain high levels of complex organic compounds such as polysaccharides and polyphenols. Because the presence of polysaccharides has been shown to inhibit enzyme activity (Fang et al., 1992; Pandey et al., 1996), it is necessary to control the contamination of these substances, which is often a cumbersome procedure.

Our recommended method for MAS of *MS1* is to extract DNA with InstaGene at the EC stage, perform one-step PCR amplification with a newly developed ING marker, and carry out genotyping by agarose gel electrophoresis. For the families with mutations of *ms1-2*, additional genotyping of the ALP marker, which can also be genotyped in a single PCR and gel electrophoresis, has been required. In any case, this combination of methods should greatly reduce the labor-intensiveness of subsequent culturing, as the results can be obtained within a few hours from a small callus sample.

## Concluding Remarks

A simple and effective method for selecting MSPs of *C. japonica* carrying the male-sterility gene *MS1*, the principal causative gene of male sterility within the mutant trees discovered to date, is described for the first time. This method uses the mutation itself as a direct marker to determine male sterility in somatic plants. This implies that male-fertile and -sterile individuals can be discriminated based on this new direct marker without the possibility of incorrectly identifying individuals. This represents a major improvement in MAS efficiency compared with the approach applied in our previous study (Maruyama et al., 2020). Furthermore, the simple methods of DNA extraction from different plant materials described here could further simplify the methodology for MAS in sugi. We believe that the methodology improved in this study will serve not only to accelerate the production of pollen-free plants but also to improve molecular breeding technology for MSPs of Japanese cedar.

## Data Availability Statement

The original contributions presented in the study are included in the article/Supplementary Materials, further inquiries can be directed to the corresponding author.

## Author Contributions

MT, TM, SU, and YM: conceptualization and methodology. YM: funding acquisition and project administration. TM: plant material preparation. MT, TM, YH, and SU: data curation and experiments and data analysis. MT and TM: writing – original draft. MT, TM, YH, SU, and YM: writing – review and editing. All authors contributed to the article and approved the submitted version.

## Funding

This research was supported by grants from the Ministry of Agriculture, Forestry and Fisheries of Japan (MAFF) and Bio-oriented Technology Research Advancement Institution (BRAIN). The Science and Technology Research Promotion Program for Agriculture, Forestry, Fisheries and Food Industry (No. 28013B) and grants from Bio-oriented Technology Research Advancement Institution (BRAIN) Research Program on Development of Innovative Technology (No. 28013BC).

## Conflict of Interest

The authors declare that the research was conducted in the absence of any commercial or financial relationships that could be construed as a potential conflict of interest.

## Publisher’s Note

All claims expressed in this article are solely those of the authors and do not necessarily represent those of their affiliated organizations, or those of the publisher, the editors and the reviewers. Any product that may be evaluated in this article, or claim that may be made by its manufacturer, is not guaranteed or endorsed by the publisher.
