# Supplementary material for: Marker-Assisted Selection for Pollen-Free Somatic Plants of Sugi (Japanese Cedar, Cryptomeria japonica): A Simple and Effective Methodology for Selecting Male-Sterile Mutants With ms1-1 and ms1-2
Source: Front Plant Sci. 2021 Oct 12;12:748110. doi: 10.3389/fpls.2021.748110 (PMC8545805; doi:10.3389/fpls.2021.748110)
Supplement: Supplementary file 1 [file Data_Sheet_1.PDF]

Supplementary Table S1. Diagnosis of male sterility for the somatic plant lines from male flower observation and genetic marker detections on ECLs, cotyledonary embryos, and developed plants.

Seed family	Cell line	Previous decision[1]	*MS1* genotype determined in the present study[2]						*MS1* diagnosis by marker genotypes	Observation of matured pollen	
			ECL	Extraction /Marker	Cotyledonary embryo	Extraction /Marker	Developed plant	Extraction /Marker		in 2020	in 2021
S3S2	SSD-9	sterile			*n.a.*	Ig[1]	*ms1-1/ms1-1*	Ct[2]	sterile	absence	absence
	SSD-18	sterile	*ms1-1/ms1-1*	Ig[1] Fc[1]	*ms1-1/ms1-1*	Ig[1]	*ms1-1/ms1-1*	Ig[1] Fc[1] Ct[2]	sterile	absence	absence
	SSD-18K	sterile	*ms1-1/ms1-1*	Ig[1]	*n.a.*	Ig[1]			sterile	absence	absence
	SSD-29	sterile	*ms1-1/ms1-1*	Ig[1]	*n.a.*	Ig[1]	*ms1-1/ms1-1*	Ig[1] Ct[2]	sterile	absence	absence
	SSD-70	sterile					*ms1-1/ms1-1*	Ct[2]	sterile	absence	absence
	SSD-73	sterile	*ms1-1/ms1-1*	Ig[1]	*ms1-1/ms1-1*	Ig[1]	*ms1-1/ms1-1*	Ig[1] Ct[2]	sterile	absence	absence
	SSD-100	sterile	*ms1-1/ms1-1*	Ig[1]	*ms1-1/ms1-1*	Ig[1]	*ms1-1/ms1-1*	Ig[1] Ct[2]	sterile	absence	absence
	SSD-111	sterile	*ms1-1/ms1-1*	Ig[1]			*ms1-1/ms1-1*	Ct[2]	sterile	absence	absence
	SSD-113	sterile	*ms1-1/ms1-1*	Ig[1]	*ms1-1/ms1-1*	Ig[1]	*ms1-1/ms1-1*	Ig[1] Ct[2]	sterile	absence	absence
	SSD-137	sterile					*ms1-1/ms1-1*	Ct[2]	sterile	absence	absence
	SSD-164	sterile	*ms1-1/ms1-1*	Ig[1]			*ms1-1/ms1-1*	Ig[1] Ct[2]	sterile	absence	absence
	SSD-168	sterile					*ms1-1/ms1-1*	Ct[2]	sterile	absence	absence
	SSD-174	sterile					*ms1-1/ms1-1*	Ct[2]	sterile	absence	absence
	SSD-182	sterile	*ms1-1/ms1-1*	Ig[1]	*ms1-1/ms1-1*	Ig[1]	*ms1-1/ms1-1*	Ig[1] Ct[2]	sterile	absence	absence
	SSD-183	sterile					*ms1-1/ms1-1*	Ct[2]	sterile	absence	absence
	SSD-195	sterile					*ms1-1/ms1-1*	Ct[2]	sterile	absence	absence
	SSD-199	sterile					*ms1-1/ms1-1*	Ct[2]	sterile	absence	absence
	SSD-270	sterile			*n.a.*	Ig[1]	*ms1-1/ms1-1*	Ct[2]	sterile	absence	absence
	SSD-272	sterile	*ms1-1/ms1-1*	Ig[1]	*ms1-1/ms1-1*	Ig[1]	*ms1-1/ms1-1*	Ig[1] Ct[1],[2]	sterile	absence	absence
	SSD-291	fertile					*Ms1/ms1-1*	Ct[1],[2]	fertile	presence	presence
	SSD-345	sterile					*ms1-1/ms1-1*	Ct[1],[2]	sterile	absence	absence
	SSD-352	sterile	*ms1-1/ms1-1*	Ig[1]	*ms1-1/ms1-1*	Ig[1]	*ms1-1/ms1-1*	Ig[1] Ct[1],[2]	sterile	absence	absence
	SSD-354	fertile					*Ms1/ms1-1*	Ct[1],[2]	fertile	presence	presence
	SSD-377	sterile					*ms1-1/ms1-1*	Ct[1],[2]	sterile	absence	absence
	SSD-383	sterile					*ms1-1/ms1-1*	Ct[1],[2]	sterile	absence	absence
	SSD-Seedling-1	--					*ms1-1/ms1-1*	Ct[2]	sterile	absence	absence
	SSD-Seedling-2	--					*Ms1/ms1-1*	Ct[2]	fertile	presence	presence
	SSD-Seedling-3	--					*ms1-1/ms1-1*	Ct[2]	sterile	absence	absence
	SSD-Seedling-4	--					*Ms1/ms1-1*	Ct[2]	fertile	presence	presence
	SSD-Seedling-5	--					*Ms1/ms1-1*	Ct[2]	fertile	presence	presence
	SSD-Seedling-6	--					*ms1-1/ms1-1*	Ct[2]	sterile	absence	absence
	Seeding-(SSD)-1	--					*ms1-1/ms1-1*	Ct[2]	sterile	absence	--
	Seedling(SSD)-2	--					*ms1-1/ms1-1*	Ct[2]	sterile	absence	--
	Seedling(SSD)-3	--					*Ms1/ms1-1*	Ct[2]	fertile	presence	--
	Seedling(SSD)-4	--					*Ms1/ms1-1*	Ct[2]	fertile	presence	--
	Seedling(SSD)-5	--					*ms1-1/ms1-1*	Ct[2]	sterile	absence	--
	Seedling(SSD)-6	--					*Ms1/ms1-1*	Ct[2]	fertile	presence	--
	SSD-Seedling-A	--					*Ms1/ms1-1*	Ct[1]	fertile	presence	presence
	SSD-Seedling-C	--					*Ms1/ms1-1*	Ct[1]	fertile	presence	presence
SS	S-3	sterile			*ms1-1/ms1-1*	Ig[1]	*ms1-1/ms1-1*	Ig[1] Ct[1]	sterile	absence	--
	S-8	fertile					*Ms1/ms1-1*	Ct[1]	fertile	--	--
	S-13	fertile					*Ms1/ms1-1*	Ct[1],[2]	fertile	presence	presence
	S-15	sterile			*ms1-1/ms1-1*	Ig[1]	*ms1-1/ms1-1*	Ig[1] Ct[1]	sterile	--	--
	S-22	doubted					*ms1-1/ms1-1*	Ig[1] Ct[1],[2]	sterile	absence	absence
	S-31	fertile					*Ms1/ms1-1*	Ct[1],[2]	fertile	presence	presence
	S-32	sterile					*ms1-1/ms1-1*	Ig[1] Ct[1]	sterile	--	--
	S-33	sterile	*ms1-1/ms1-1*	Ig[1]			*ms1-1/ms1-1*	Ct[1],[2]	sterile	absence	absence
	S-42	fertile					*Ms1/ms1-1*	Ct[1],[2]	fertile	presence	presence
	S-43	fertile					*Ms1/ms1-1*	Ct[1],[2]	fertile	presence	presence
	S-48	fertile					*Ms1/ms1-1*	Ig[1] Ct[1]	fertile	presence	--
	**S-55**	**fertile**			*ms1-1/ms1-1*	Ig[1]	*ms1-1/ms1-1*	Ig[1] Ct[1],[2]	sterile	absence	absence
	S-56	fertile					*Ms1/ms1-1*	Ct[1],[2]	fertile	presence	presence
	S-58	sterile	*ms1-1/ms1-1*	Ig[1]	*ms1-1/ms1-1*	Ig[1]	*ms1-1/ms1-1*	Ct[1],[2]	sterile	absence	absence
	S-61	sterile					*ms1-1/ms1-1*	Ct[1],[2]	sterile	absence	absence
	S-62	fertile					*Ms1/ms1-1*	Ct[1],[2]	fertile	presence	presence
	S-64	fertile	*Ms1/ms1-1*	Ig[1]	*Ms1/ms1-1*	Ig[1]	*Ms1/ms1-1*	Ig[1] Ct[1],[2]	fertile	presence	presence
	S-70	sterile					*ms1-1/ms1-1*	Ct[1]	sterile	--	--
	S-75	fertile					*Ms1/ms1-1*	Ct[1],[2]	fertile	presence	presence
	S-79	fertile					*Ms1/ms1-1*	Ct[1],[2]	fertile	presence	presence
	S-85	sterile	*ms1-1/ms1-1*	Ig[1]	*ms1-1/ms1-1*	Ig[1]	*ms1-1/ms1-1*	Ct[1],[2]	sterile	absence	absence
	S-86	sterile					*ms1-1/ms1-1*	Ct[1],[2]	sterile	absence	absence
	S-88	fertile					*Ms1/ms1-1*	Ct[1]	fertile	presence	--
	S-89	fertile					*Ms1/ms1-1*	Ct[1],[2]	fertile	presence	presence
	S-90	sterile					*ms1-1/ms1-1*	Ct[1]	sterile	--	--
	S-100	fertile					*Ms1/ms1-1*	Ct[1],[2]	fertile	presence	presence
	S-106	sterile			*ms1-1/ms1-1*	Ig[1]	*ms1-1/ms1-1*	Ct[1],[2]	sterile	absence	absence
	S-114	sterile					*ms1-1/ms1-1*	Ct[1],[2]	sterile	absence	absence
	S-115	fertile					*Ms1/ms1-1*	Ct[1]	fertile	presence	--
	S-116	sterile					*ms1-1/ms1-1*	Ct[1],[2]	sterile	absence	absence
	S-119	fertile					*Ms1/ms1-1*	Ct[1],[2]	fertile	presence	presence
	S-123	sterile					*ms1-1/ms1-1*	Ct[1]	sterile	--	--
	S-129	sterile	*ms1-1/ms1-1*	Ig[1]	*ms1-1/ms1-1*	Ig[1]			sterile	--	--
	S-130	sterile			*ms1-1/ms1-1*	Ig[1]	*ms1-1/ms1-1*	Ct[1],[2]	sterile	absence	--

	S-132	fertile				*Ms1/ms1-1*	Ig[1]	*Ms1/ms1-1*			Ct[1],[2]	fertile	presence	presence
	S-139	sterile	*ms1-1/ms1-1*	Ig[1]		*ms1-1/ms1-1*	Ig[1]					sterile	--	--
	S-140	sterile				*ms1-1/ms1-1*	Ig[1]	*ms1-1/ms1-1*	Ig[1]	Fc[1]	Ct[1]	sterile	absence	absence
	S-142	sterile						*ms1-1/ms1-1*			Ct[1],[2]	sterile	absence	absence
	S-146	fertile						*Ms1/ms1-1*			Ct[1],[2]	fertile	presence	presence
	S-148	sterile				*ms1-1/ms1-1*	Ig[1]	*ms1-1/ms1-1*			Ct[1],[2]	sterile	absence	absence
	S-151	sterile	*ms1-1/ms1-1*	Ig[1]		*ms1-1/ms1-1*	Ig[1]	*ms1-1/ms1-1*			Ct[1],[2]	sterile	absence	absence
	Seed-(S)-1	--						*Ms1/ms1-1*			Ct[2]	fertile	presence	--
	Seed-(S)-2	--						*Ms1/ms1-1*			Ct[2]	fertile	presence	--
	Seed-(S)-3	--						*ms1-1/ms1-1*			Ct[2]	sterile	absence	--
	Seed-(S)-4	--						*Ms1/ms1-1*			Ct[2]	fertile	presence	--
	Seed-(S)-5	--						*ms1-1/ms1-1*			Ct[2]	sterile	absence	--
	Seed-(S)-6	--						*Ms1/ms1-1*			Ct[2]	fertile	presence	--
FSKam	FSKam-22	sterile						*ms1-1/ms1-1*			Ct[1],[2]	sterile	absence	absence
	FSKam-43	sterile	*ms1-1/ms1-1*	Ig[1]	Fc[1]			*ms1-1/ms1-1*	Ig[1]		Ct[1],[2]	sterile	absence	absence
	FSKam-53	fertile						*Ms1/ms1-1*			Ct[1],[2]	fertile	presence	presence
	FSKam-54	fertile						*Ms1/ms1-1*			Ct[1],[2]	fertile	presence	presence
	FSKam-59	sterile						*ms1-1/ms1-1*			Ct[1],[2]	sterile	absence	absence
	FSKam-61	sterile	*ms1-1/ms1-1*	Ig[1]								sterile	--	--
	FSKam-65	sterile	*ms1-1/ms1-1*	Ig[1]								sterile	--	--
	FSKam-72	fertile	*Ms1/ms1-1*	Ig[1]								fertile	--	--
	FSKam-133	fertile	*Ms1/ms1-1*	Ig[1]	Fc[1]			*Ms1/ms1-1*	Ig[1]	Fc[1]	Ct[1],[2]	fertile	presence	presence
	FSKam-136	fertile						*Ms1/ms1-1*	Ig[1]		Ct[1],[2]	fertile	presence	presence
FO7	FO7-19	sterile						*ms1-1/ms1-2*	Ig[1]	Fc[1]	Ct[1],[2],[3]	sterile	absence	absence
	FO7-23	sterile						*ms1-1/ms1-2*	Ig[1]	Fc[1]	Ct[1],[2],[3]	sterile	absence	absence
	FO7-33	fertile						*Ms1/ms1-1*		Fc[1]	Ct[1],[2],[3]	fertile	presence	presence
	FO7-71	sterile						*ms1-1/ms1-2*			Ct[1],[2],[3]	sterile	absence	absence
	FO7-75	sterile						*ms1-1/ms1-2*		Fc[1]	Ct[1],[2],[3]	sterile	absence	absence
	FO7-97	sterile						*ms1-1/ms1-2*	Ig[1]	Fc[1]	Ct[1],[2],[3]	sterile	absence	absence
	FO7-141	sterile	*ms1-1/ms1-2*	Ig[1],[3]	Fc[1]	*Ms1/ms1-1*	Ig[1]	*ms1-1/ms1-2*	Ig[1]	Fc[1]	Ct[1],[2],[3]	sterile	absence	absence
	FO7-144	sterile	*ms1-1/ms1-2*	Ig[1],[3]	Fc[1]	*Ms1/ms1-1*	Ig[1]					sterile	--	--
FSKas	FSKas-9	fertile	*Ms1/ms1-1*	Ig[1]	Fc[1]			*Ms1/ms1-1*	Ig[1]	Fc[1]	Ct[1],[2]	fertile	presence	presence
	FSKas-28	fertile						*Ms1/ms1-1*			Ct[1],[2]	fertile	presence	presence
	FSKas-72	sterile	*ms1-1/ms1-1*	Ig[1]								sterile	--	--
	FSKas-78	sterile	*ms1-1/ms1-1*	Ig[1]	Fc[1]			*ms1-1/ms1-1*	Ig[1]	Fc[1]	Ct[1],[2]	sterile	absence	absence
	FSKas-85	sterile	*ms1-1/ms1-1*	Ig[1]								sterile	--	--
	FSKas-122	sterile						*ms1-1/ms1-1*			Ct[1],[2]	sterile	absence	absence
TOS	TOS-3-31	sterile						*ms1-1/ms1-1*			Ct[1],[2]	sterile	absence	absence
	TOS-1-58	fertile	*Ms1/ms1-1*	Ig[1]		*Ms1/ms1-1*	Ig[1]	*Ms1/ms1-1*	Ig[1]	Fc[1]	Ct[1],[2]	fertile	presence	presence
	TOS-1-6	sterile						*ms1-1/ms1-1*			Ct[1],[2]	sterile	absence	absence
Non-*ms1* family	T2-14-1	fertile						*Ms1/Ms1*			Ct[1]	fertile	presence	--
	T2-14-149	fertile						*Ms1/Ms1*			Ct[1]	fertile	presence	--
	T4-4-1	fertile						*Ms1/Ms1*			Ct[1]	fertile	presence	--
	T4-11-51	fertile	*Ms1/Ms1*	Ig[1]	Fc[1]	*n.a.*	Ig[1]	*Ms1/Ms1*	Ig[1]	Fc[1]	Ct[1]	fertile	presence	presence

[1]*MS1* diagnosis using marker closely linked to *MS1* on ECLs (Maruyama et al., 2020).

[2]DNA extraction methods, Ig: InstaGene, Fc: FTA Card, Ct: simplified CTAB method, and genetic markers: [1] Indel genotyping (ING) marker (this study), [2] allele-specific PCR (ASP) markers for *MS1* and *ms1-1* (Hasegawa et al., 2020), and [3] amplified length polymorphism (ALP) marker for *ms1-2* genotyping (Hasegawa et al., 2020). *n.a.* : not amplified.
