# Supplementary material for: Marker-Assisted Selection for Pollen-Free Somatic Plants of Sugi (Japanese Cedar, Cryptomeria japonica): A Simple and Effective Methodology for Selecting Male-Sterile Mutants With ms1-1 and ms1-2
Source: Front Plant Sci. 2021 Oct 12;12:748110. doi: 10.3389/fpls.2021.748110 (PMC8545805; doi:10.3389/fpls.2021.748110)
Supplement: Supplementary file 2 [file Data_Sheet_2.pdf]

Supplementary Material

1 Supplementary Table

Supplementary Table S1. Diagnosis of male sterility for the somatic plant lines from male flower observation and genetic marker detection on ECLs, cotyledonary embryos, and developed plants.

Excel file.

2 Supplementary Figures

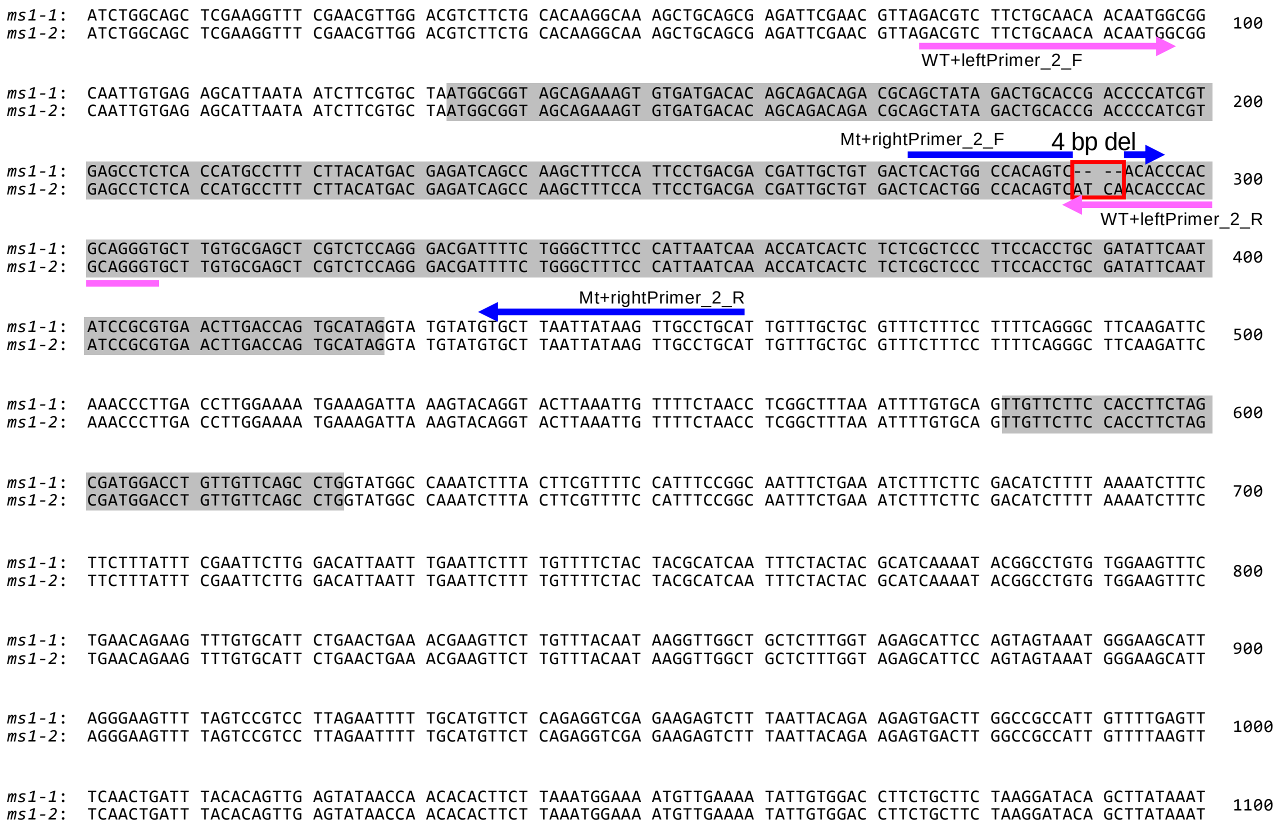

Supplementary Figure S1. Mutation sites causative of *MALE STERILITY 1* (4-bp del and 30-bp del of red frame) and the designed location of the markers that distinguish them. Primers for the ING marker (WT+leftPrimer_2 and mut+leftPrimer_2) and LAMP reaction (FIP, BIP, F3, B3, LF, LB, and PNAs) are shown on the alignment of the allele sequences of the CJt020762 gene of 'Fukushima-funen 1' (*ms1-1*: LC536580) and 'Ōi 7' (*ms1-2*: LC538205). The bases shaded in gray represent CDS.

```
ms1-1:  TCTTTAATTT AGAAAAGCAA CAAGTGCAAC CTATAGACAC AGAAGGTCCT TGGATTCCAT TTGAAAAAAA ATTGAAGGGC GCCAGAGAGG TCTTCGGTTC
ms1-2:  TCTTTAATTT AGAAAAGCAA CAAGTGCAAC CTATAGACAC AGAAGGTCCT TGGATTCCAT TTGAAAAAAA ATTGAAGGGC GCCAGAGAGG TCTTCGGTTC   1200

ms1-1:  GAGTTCTTAT TTAAAATTAC AAAAAATTAA TGTGTTCCGT AGAAATTTAA CATTCAAGCC CTAATTTTGC ACCATATTTC TTGAATCTTC AAATTACTTG
ms1-2:  GAGTTCTTAT TTAAAATTAC AAAAAATTAA TGTGTTCCGT AGAAATTTAA CATTCAAGCC CTAATTTTGC ACCATATTTC TTGAATCTTC AAATTACTTG   1300

ms1-1:  CCTTCGAAAT GAATCTTTTG TATACTAAGA ATAAAATCCA GCTGTTCTTT TCACGTTTTA CATTGTTTGG GTTTAAATCA ACGTTCTTTG CGTAATTGGT
ms1-2:  CCTTCGAAAT GAATCTTTTG TATACTAAGA ATAAAATCCA GCTGTTCTTT TCACGTTTTA CATTGTTTGG GTTTAAATCA ACGTTCTTTG CGTAATTGGT   1400

ms1-1:  CTCAGGCTCG AATCCCCCAC CGCCTGTAGT ATATGGGAAT CATTAAGGAG GTTTTGGTTT GTGCTTAATT AAGAAAGCAA TAAGTGCAAC CTATACACAC
ms1-2:  CTCAGGCTCG AATCCCCCAC CGCCTGTAGT ATATGGGAAT CATTAAGGAG GTTTTGGTTT GTGCTTAATT AAGAAAGCAA TAAGTGCAAC CTATACACAC   1500

ms1-1:  AGGAGGTCCT ACGATTCCGT CAAAAGAAAA ACTGAAGCGA ATCTCAGAGG TCTTCGGTTC GAATCCCAGA GGTATGGAGC ACCCCATACA CATTGCAGGC
ms1-2:  AGGAGGTCCT ACGATTCCGT CAAAAGAAAA ACTGAAGCGA ATCTCAGAGG TCTTCGGTTC GAATCCCAGA GGTATGGAGC ACCCCATACA CATTGCAGGC   1600

ms1-1:  TCTGATCCGT AGAAATTTAG CACTCAAGCC CTAGTTTTGC ACCATATTTC TAAGCCCCAA GATTCATTAA CTGTTCTTTT CATGTTTTAC CGTTGGTCTT
ms1-2:  TCTGATCCGT AGAAATTTAG CACTCAAGCC CTAGTTTTGC ACCATATTTC TAAGCCCCAA GATTCATTAA CTGTTCTTTT CATGTTTTAC CGTTGGTCTT   1700

ms1-1:  CTTGGATTTT AAAATTTCTT GCCTCCTGAA TGAATCTTTT GTATCCCATG CATGAAATCT TAACTGTTTT TTTCACGTTT TACACCGTTG ATGTTAAAAA
ms1-2:  CTTGGATTTT AAAATTTCTT GCCTCCTGAA TGAATCTTTT GTATCCCATG CATGAAATCT TAACTGTTTT TTTCACGTTT TACACCGTTG ATGTTAAATA   1900

ms1-1:  CTAATGTTCT TTGCGTAATC GGTCTCAGGC TCGAATCCAC CGCCACGACT ATTTCATCCC GAAGCTCCAT CGCCGGCGCT TGAGGCTGTT CCTCCGGTGC
ms1-2:  CTAATGTTCT TTGCGTAATC GGTCTCAGGC TCGAATCCAC CGCCACGACT ATTTCATCCC GAAGCTCCAT CGCCGGCGCT TGAGGCTGTT CCTCCGGTGC   2000

ms1-1:  TTGAAGCTCC GCCAATGGAG AGCGTTCCCG ATCAGCCGGA TGATTCGCCC TTTCCAAATG TTAGCATTGC TGGAGCCCTT TTCACGCAAT CTTTATTACG
ms1-2:  TTGAAGCTCC GCCAATGGAG AGCGTTCCCG ATCAGCCGGA TGATTCGCCC TTTCCAAATG TTAGCATTGC TGGAGCCCTT TTCACGCAAT CTTTATTACG   2100
                    PNA_WT1        F3      30 bp del      FIP (F2)     PNA_WT2        LF
ms1-1:  GCTATTTTGG GGATCACTGC TAACCGCCAT TTTAACTGTC GGTTATTTGC ATTGAAGTTT GATACACCGG AGGTTTAAAA CTTTAACATA TTTCTTCTTT
ms1-2:  GCTATTTTGG GGATCAC--- ---------- ---------- -------TGC ATTGAAGTTT GATACACCGG AGGTTTAAAA CTTTAACATA TTTCTTCTTT   2200
            FIP (F1c)                                               BIP (B1c)                 LB
ms1-1:  TGCCATGGAA ACAGAGTTCA AAGTTATATT TAGCAGTGAA TGAATTGCTG TTGTCTGTAT TCCGAATTTT GAGACACTTC TCTGCAATCA ATGTTACATA
ms1-2:  TGCCATGGAA ACAGAGTTCA AAGTTATATT TAGCAGTGAA TGAATTGCTG TTGTCTGTAT TCCGAATTTT GAGTAACTTC TCTGCAATCA ATGTTACATA   2300
                  BIP (B2)                                 B3

ms1-1:  TATATCTGTT ATGGCTAA…
ms1-2:  TATATCTGTT ATGGCTAA…
```

Supplementary Figure S1. Continued.

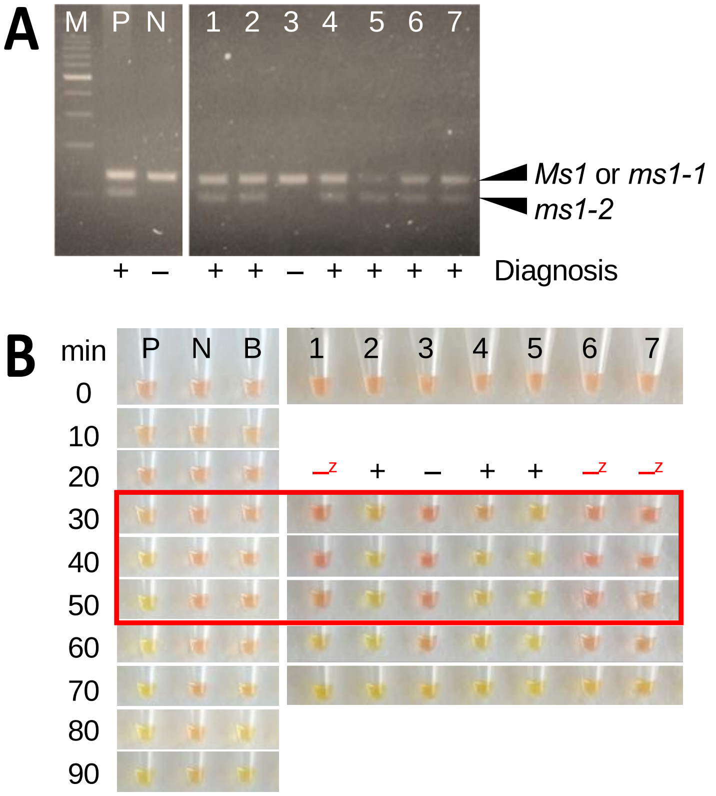

Supplementary Figure S2. Diagnosis of the presence of *ms1-2* allele (+: presence, –: absent) using the amplified length polymorphic (ALP) marker (**A**) and LAMP reaction (**B**). M: 100 bp ladder marker, P: positive sample, 'Ōi 7' (*Ms1/ms1-2*), N: negative sample, 'Suzu 2' (*Ms1/ms1-1*), B: blank control (H_2O), 1–8: crude DNA extracts of offspring of FO7 (1: FO7-19, 2: FO7-23, 3: FO7-33, 4: FO7-71, 5: FO7-75, 6: FO7-97, 7: FO7-141). [z]false-negative decisions.
